# Supplementary material for: Long-read genome sequencing improves detection and functional interpretation of structural and repeat variants in autism
Source: Cell Genom. 2026 Mar 9;6(5):101186. doi: 10.1016/j.xgen.2026.101186 (PMC13174233; doi:10.1016/j.xgen.2026.101186)
Supplement: Document S1. Figures S1–S19 [file mmc1.pdf]

Cell Genomics, Volume *6*

Supplemental information

Long-read genome sequencing improves detection and functional interpretation of structural and repeat variants in autism

Milad Mortazavi, James Guevara, Joshua Diaz, Stephen Tran, Helyaneh Ziaei Jam, Chloe Reeves, Sergey Batalov, Kristen Jepsen, Matthew Bainbridge, Aaron D. Besterman, Melissa Gymrek, Abraham A. Palmer, and Jonathan Sebat

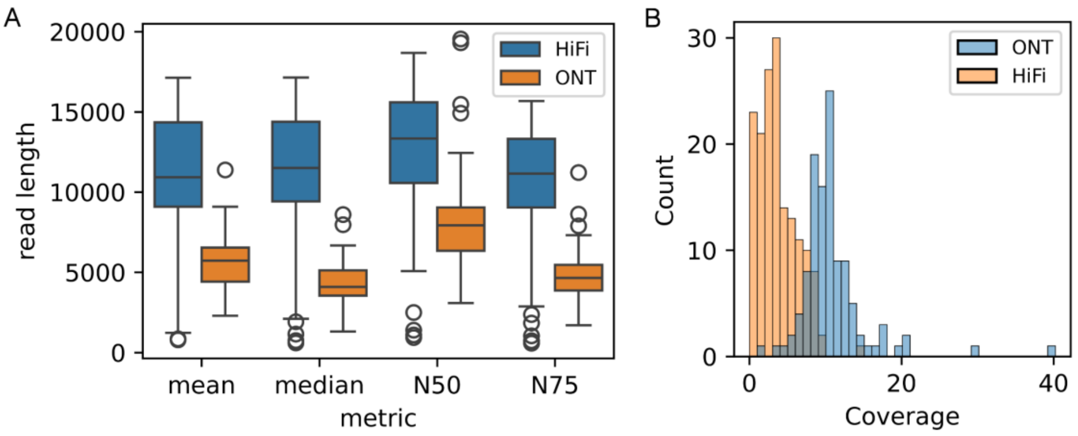

Figure S1. Read length and coverage distribution of subjects in the cohort. Related to STAR methods.
(A) Read length distribution stratified by platform. The box borders represent the first and third quartiles (Q1 and Q3), the box height is the interquartile range (IQR=Q3-Q1), and the middle line represents the median. The top whisker shows the last data point which is still less than Q3+1.5xIQR, and the bottom whisker shows the last data point which is still larger than Q1-1.5xIQR **(B)** Coverage distribution of subjects in the cohort stratified by platform.

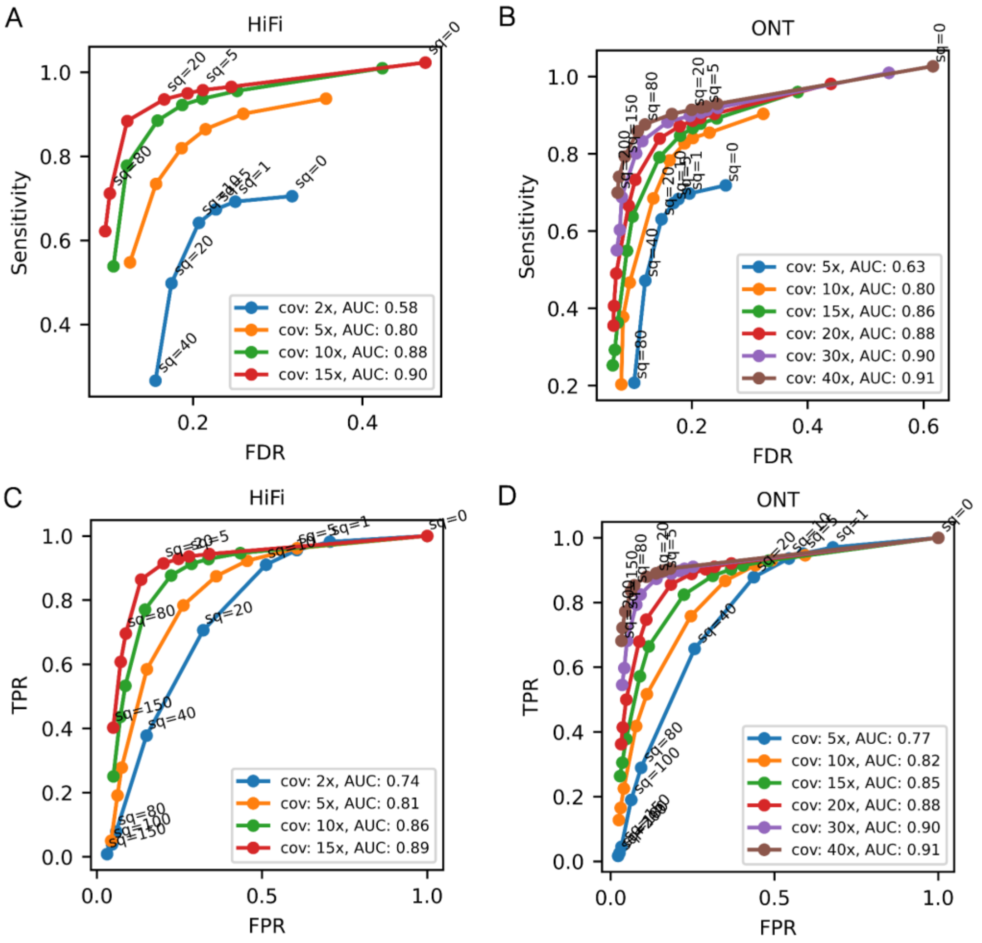

Figure S2. Benchmarking snoopSV (an in-house genotyping method) to detect allele depth (AD) and assign quality metrics to SVs. Related to STAR methods.

(A) Sensitivity vs. False Discovery Rate (FDR) for HiFi data. **(B)** Sensitivity vs. False Discovery Rate (FDR) for ONT data. **(C)** True Positive Rate (TPR) versus False Positive Rate (FPR) for HiFi data. **(D)** True Positive Rate (TPR) versus False Positive Rate (FPR) for ONT data. Area Under the Curve (AUC) for each curve is annotated in the figures.

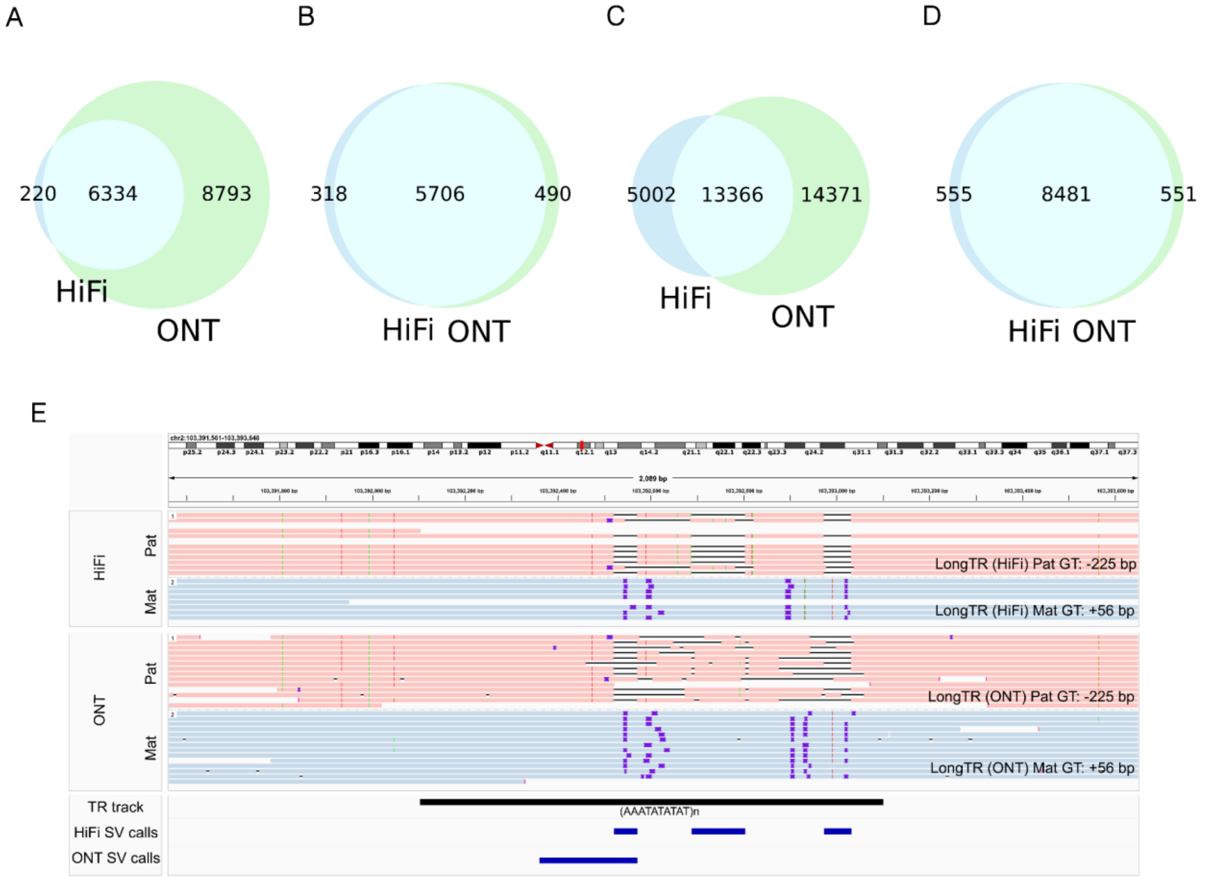

Figure S3. SV and TR concordance for REACH000236 between HiFi (15x) and ONT (40x) WGS data. Related to STAR methods.
(A) Non-TR SVs detected by *Sniffles2* for REACH000236 before merging with other samples with 2 supporting reads show 41% concordance. **(B)** Non-TR SVs of REACH000236 after merging with other samples using SQ≥20 shows 88% concordance. **(C)** TR SVs detected by *Sniffles2* for REACH000236 before merging with other samples with 2 supporting reads show 41% concordance. **(D)** TR SVs genotyped by LongTR for REACH000236 show 88% concordance. **(E)** An example of a TR region where alignment is noisy and introduces scattered insertions and deletions for both ONT and HiFi data. The insertions/deletions are scattered across the TR region and SV calling needs special attention.

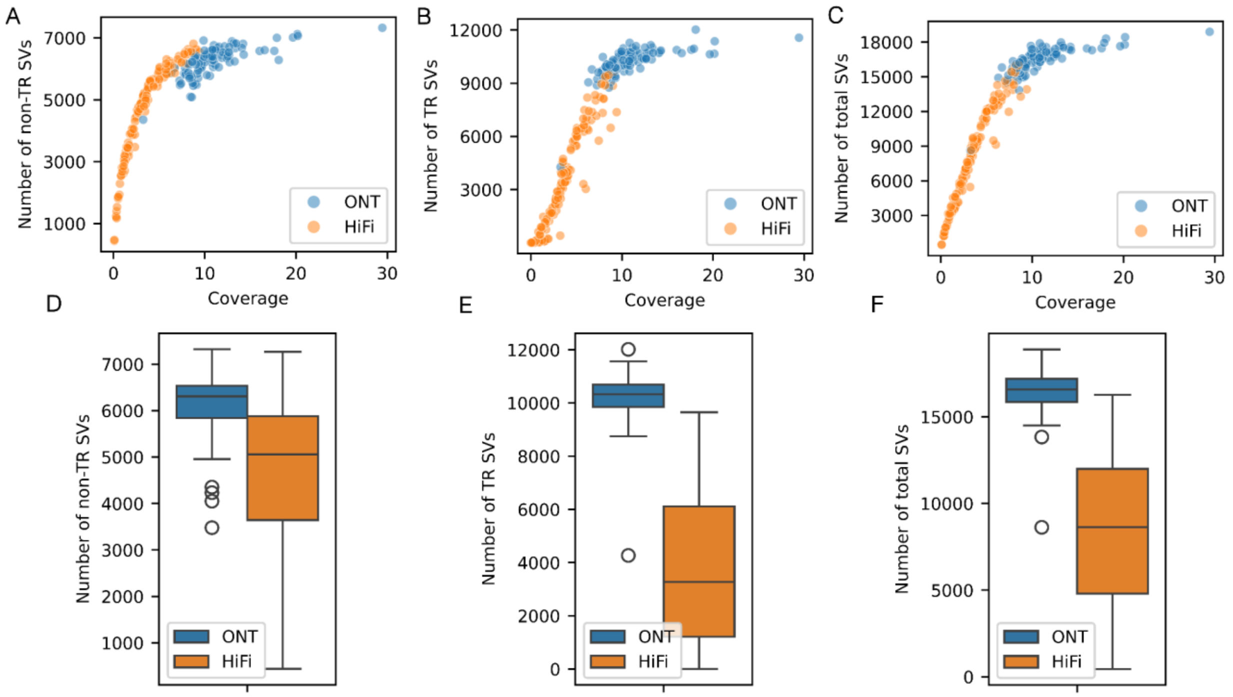

Figure S4. Number of structural variations found for each subject as a function of sequencing coverage and stratified by platform. Related to STAR methods.
(A,D) Number of non-TR SVs with sample quality greater than 20. **(B,E)** Number of TR regions with at least 50 bp deviation, at least two supporting reads and genotyping quality greater than 0.9. **(C,F)** Total number of SVs in non-TR and TR regions.

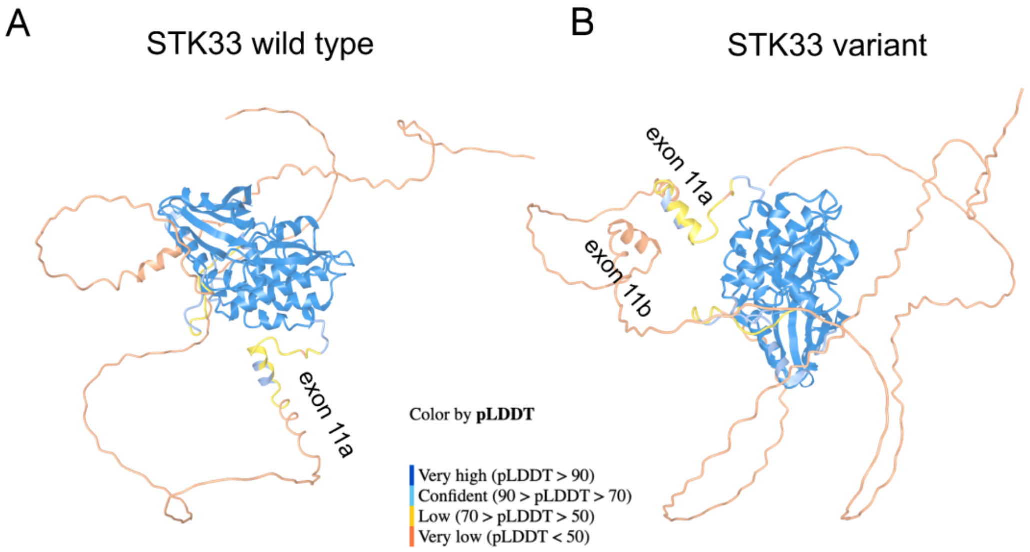

Figure S5. STK33 protein structure with and without mutation colored by pLDDT score. Related to Figure 2.
(A) Wild-type STK33 protein. **(B)** Mutant STK33 protein with exon 11 duplication.

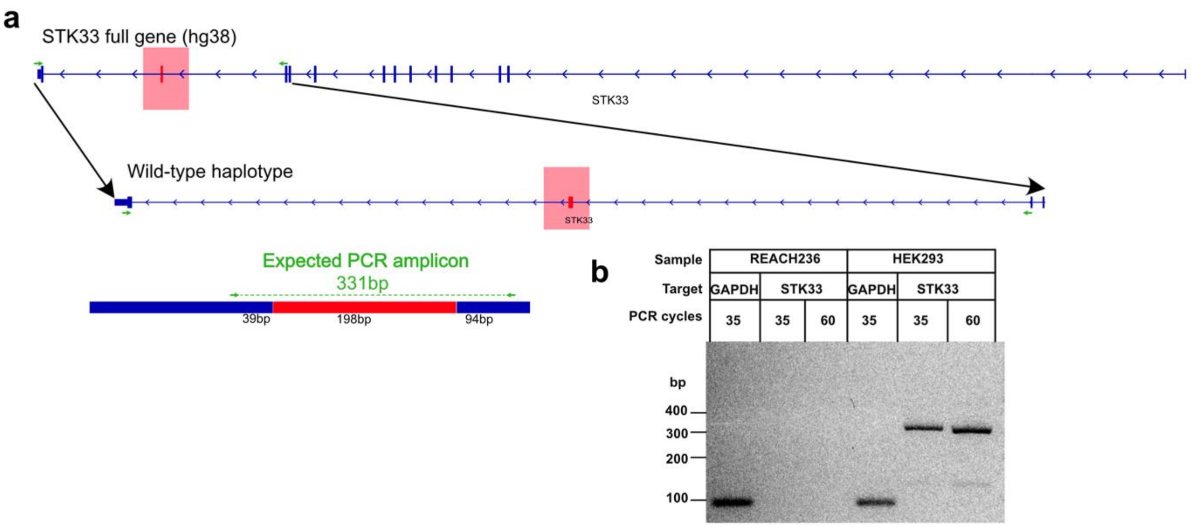

Figure S6. Polymerase chain reaction of human whole blood for the *STK33* WT/duplicated exon. Related to Figure 2.
(A) Schematic depicting forward and reverse primers (green arrows) targeting the penultimate exon (highlighted and boxed red) of *STK33* in human hg38. Top track is the *STK33* full gene transcript from RefSeq. Lower tracks show the penultimate exon targeted by PCR primers and the expected amplicon that is expected to be generated from spliced *STK33* RNA.
(B) Gel electrophoresis of PCR run on a human healthy whole blood sample (REACH000236). Lane 1: GAPDH positive control shows expected 96 bp amplicon. Lanes 2-3: *STK33* failed to amplify at either 35 PCR cycles (Lane 2) or 60 PCR cycles (Lane 3). Lanes 4-6: Positive control HEK293 cDNA sample. PCR shows expected GAPDH (Lane 4) and *STK33* (Lanes 5-6) bands. Annotations above lanes list the sample, target gene, number of PCR cycles.

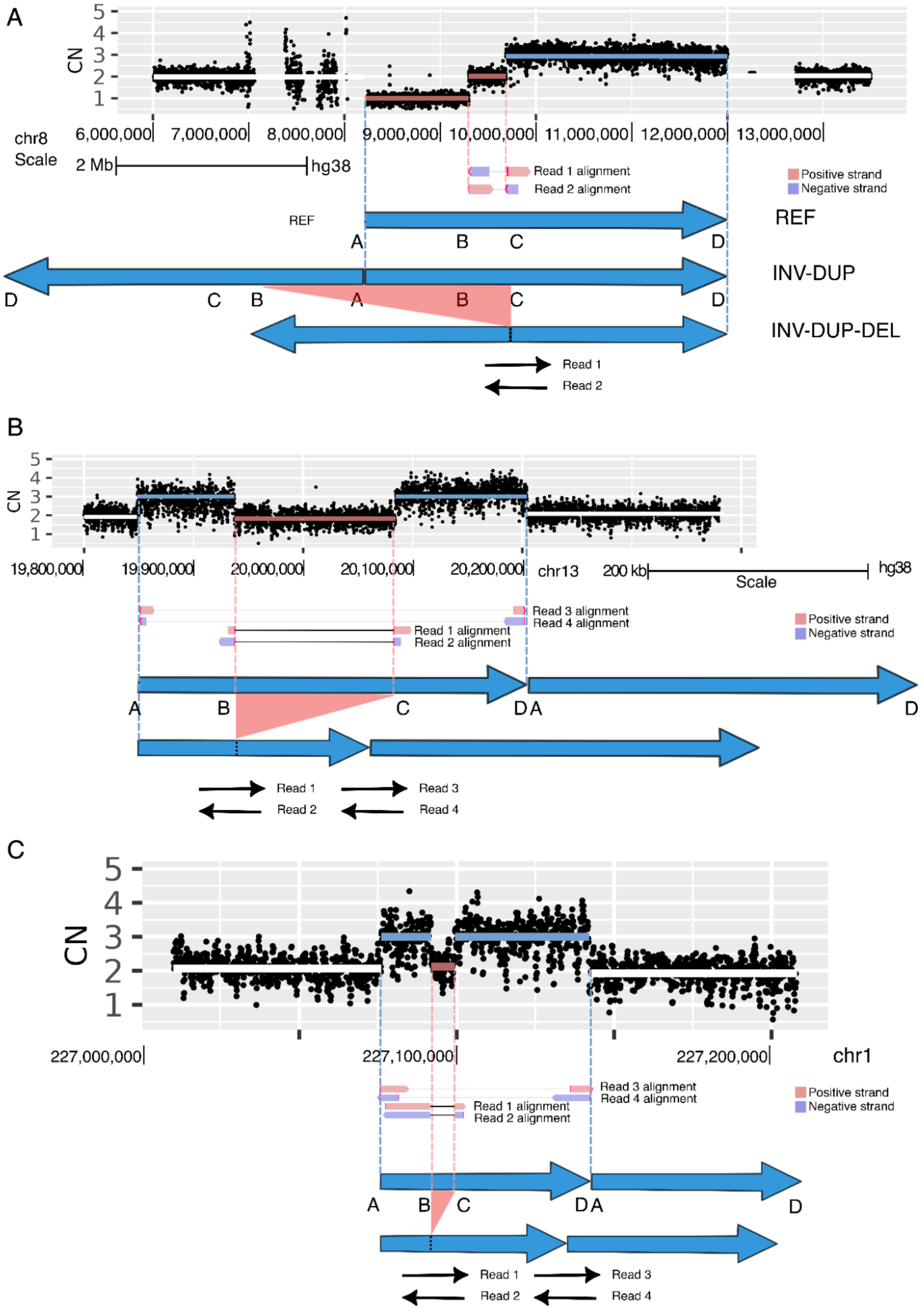

Figure S7. Split long reads passing deletion/duplication junctions support presence of complex SV breakpoints. Related to Figure 3.
(A) Corresponding to the complex SV in Figure 3A. Read 1 and 2 passing the inversion junction generate split alignments between B-C in the reference genome. **(B)** Corresponding to the complex SV in Figure 3C. Reads spanning the deletion and duplication junctions generate split alignments between B-C and A-D in the reference genome. **(C)** Corresponds to the complex SV in Figure 3E. Reads spanning the deletion and duplication junctions generate split alignments between B-C and A-D in the reference genome.

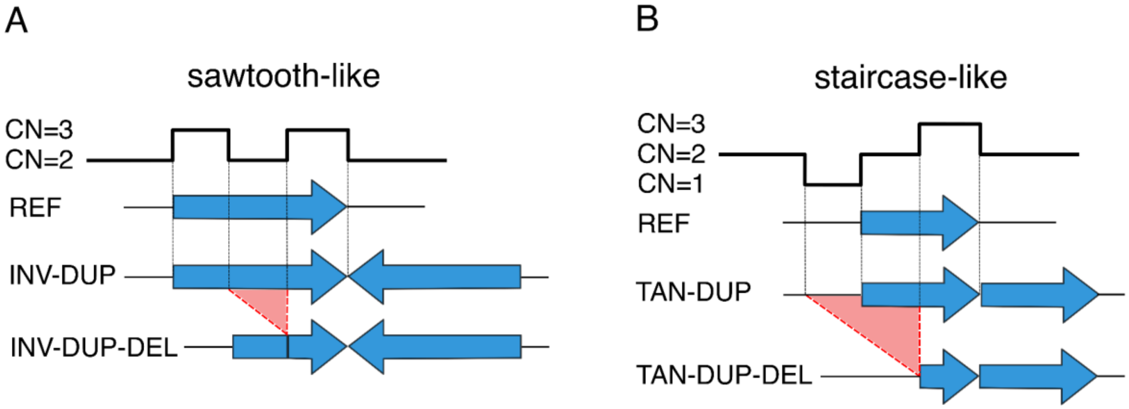

Figure S8. Schematics of complex rearrangements not observed in the call set; however, they generate coverage profiles similar to the ones observed. Related to Figure 3.
(A) Sawtooth-like coverage profile pattern is generated as a result of a nested inverted duplication and a deletion. **(B)** Staircase-like coverage profile pattern is generated as a result of a tandem duplication and a deletion spanning the breakpoint of the duplication.

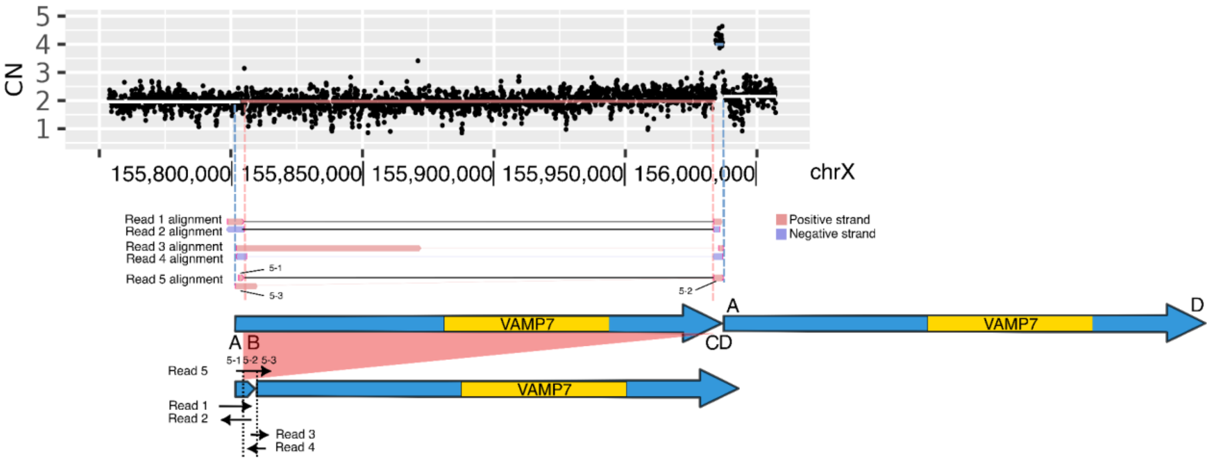

Figure S9. A monomorphic 2.7 kb insertion could be explained by a complex DUP-DEL rearrangement, and may also represent an error in the grch38 reference genome. Related to Figure 3.
In addition to the 3 examples of DUP-DEL SVs shown in Figure 3, we detected a 4th DUP-DEL signature representing a duplication (chrX:155,803,260-155,987,250) and a nested deletion (chrX:155,803,824-155,983,780) that reverts all but 2.7 kb of sequence of the duplication on the left, and has a neutral effect on the copy number of the gene *VAMP7*. However, this SV was monomorphic in our sample (allele frequency 100%). A lack of this DUP-DEL in the grch38 reference may represent a rare ancestral allele or an error in the grch38 reference in which the duplicated sequences were not correctly assembled. The same SV is represented as a 2.7 kb insertion in gnomAD v4.1 (INS_CHRX_D6524659) with frequency of 0.67. Since we have yet to find an allele that matches the reference, we suspect that this SV is not a common structural polymorphism in the population, but is actually a complex DUP-DEL event that is at or close to fixation in humans. The copy number from Illumina WGS is shown for one sample REACH000626.

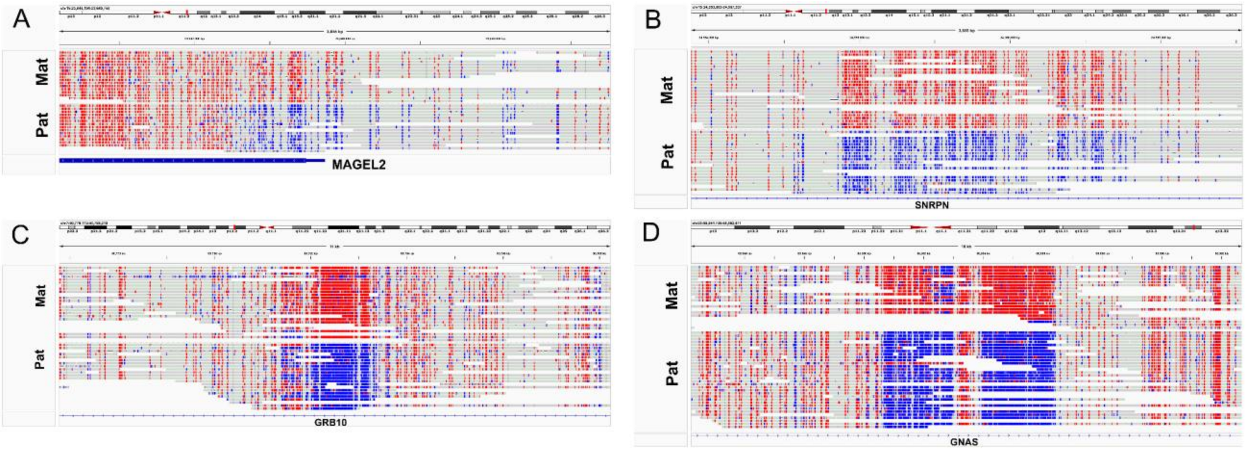

Figure S10. LR-WGS reveals methylation bias in maternal and paternal haplotypes in Imprinted SFARI genes. Related to Figure 4.
(A-D) LR-WGS methylation signature in four imprinted SFARI genes: *MAGEL2, SNRPN, GRB10* and *GNAS* ([S1], [S2]) for REACH000236 with ONT data.

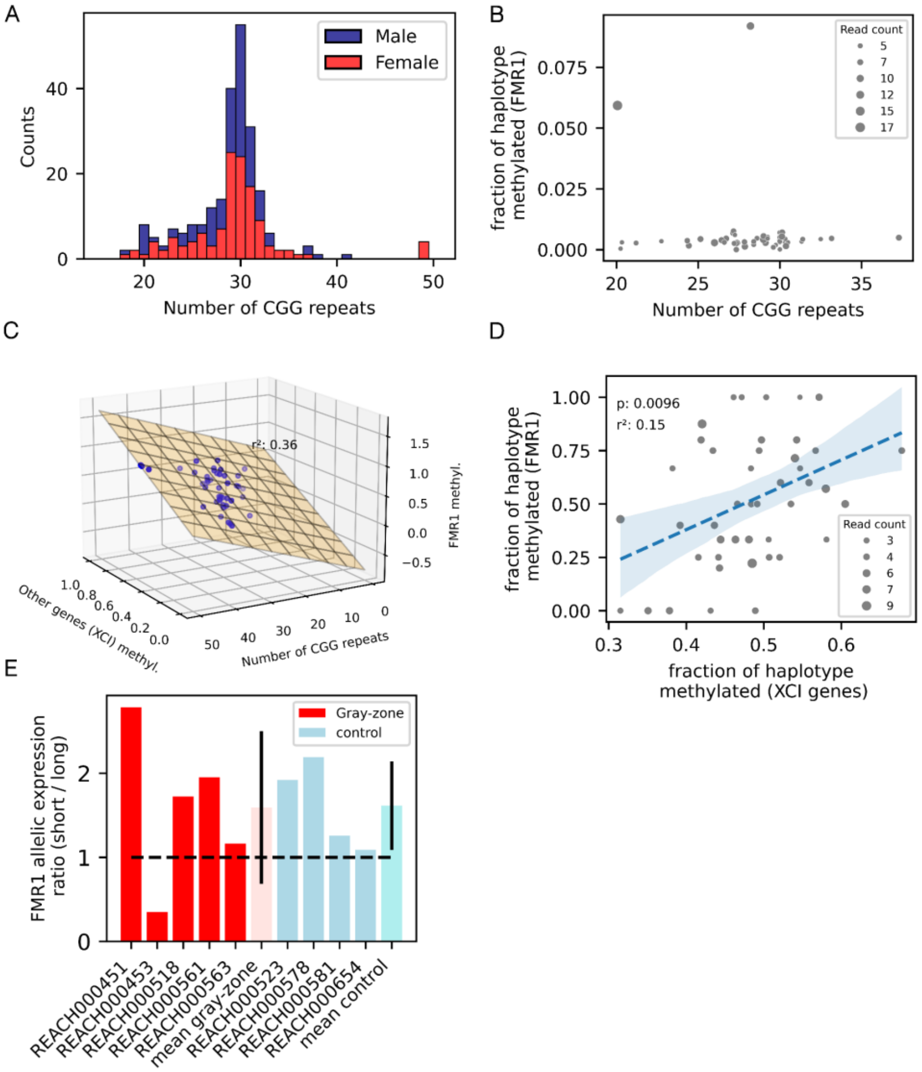

Figure S11. Details about the CGG repeat size and its effects on the methylation of the *FMR1* promoter. Related to Figure 5.

(A) Distribution of the number of CGG repeats in the *FMR1* 5'UTR in the cohort. **(B)** Fraction of haplotype methylated at 5'UTR region of *FMR1* for male subjects.The dots at y=0 are jittered to avoid overlapping. **(C)** Ordinary least squares model of the fraction of haplotype methylated in *FMR1* 5'UTR region as a function of CGG repeat length and average fraction of haplotypes methylated for other X chromosome genes (representing XCI) for females. **(D)** Ordinary least squares model of fraction of haplotype methylated in *FMR1* 5'UTR region as a function of average fraction of haplotypes methylated for other X chromosome genes (representing XCI) for females. The size is proportional to the number of reads in the haplotype at the *FMR1* 5'UTR. **(E)**: Allelic RNA-seq expression ratio of short over long CGG haplotypes for five gray-zone and four control subjects (**Table S13**). The arithmetic mean for each group is plotted with whiskers representing the standard deviation in each group.

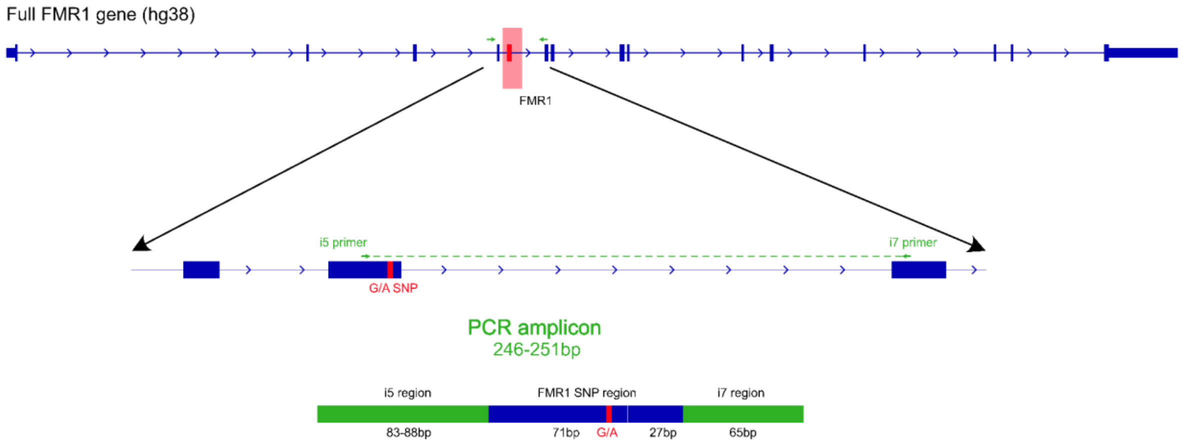

Figure S12. Polymerase chain reaction of human whole blood for allele specific mRNA expression of *FMR1* SNP. Related to Figure 5.
Schematic depicting i5 forward and i7 reverse primers (green arrows) targeting the 4th-6th exon (highlighted and boxed red) of *FMR1* in human hg38. Top track is the *FMR1* full gene transcript from RefSeq. Lower tracks show the 4th-6th exon targeted by PCR primers and an expected amplicon that is expected to be generated from spliced *FMR1* mRNA.

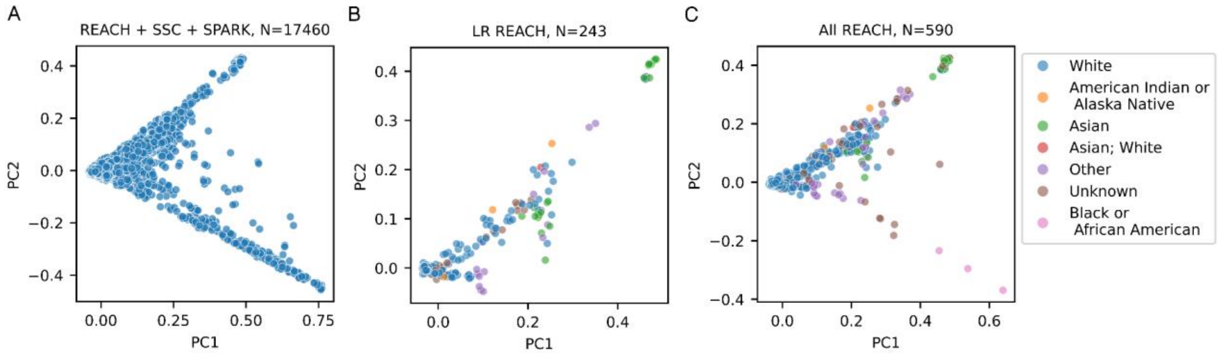

Figure S13. First two principal components (PCs) derived from SNV data. Related to STAR methods.
(A) Combination of REACH, SSC and SPARK cohorts. **(B)** Subjects participating in the LR-WGS REACH cohort; **(C)** All subjects in the REACH cohort.

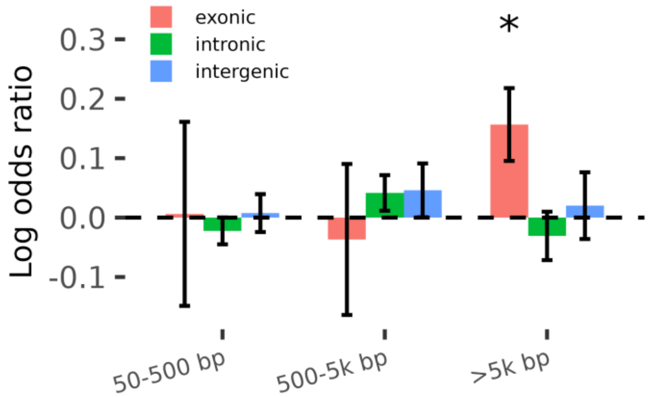

Figure S14. SV burden association with ASD case status stratified by SV size and functional consequence. Related to Figure 6.
No association is observed in the intronic and intergenic categories, and the only association observed is in the large and exonic SVs. Asterisks indicate statistical significance (*p* between 0.01 and 0.05), and error bars represent 95% confidence intervals.

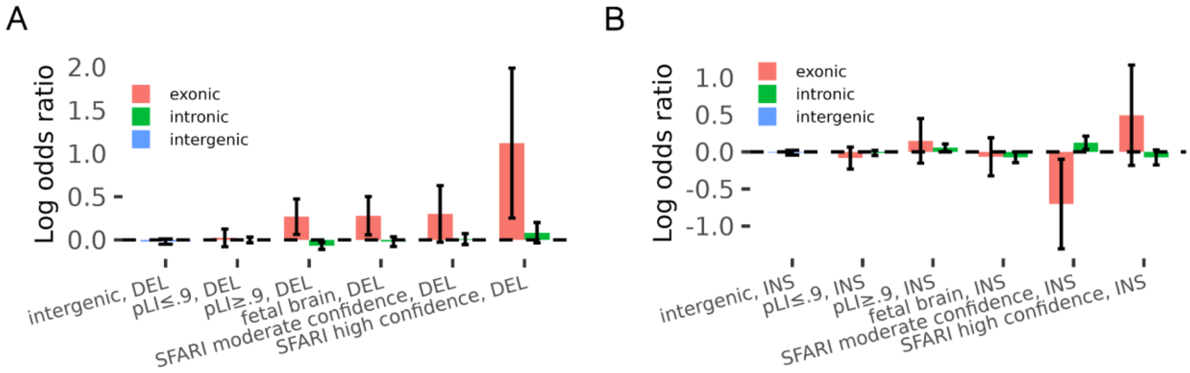

Figure S15. Stratified deletion and duplication/insertion burden test associations for SVs. Related to Figure 6.
(A) Deletion burden association tests. **(B)** Insertion/duplication burden association tests. Error bars represent 95% confidence intervals.

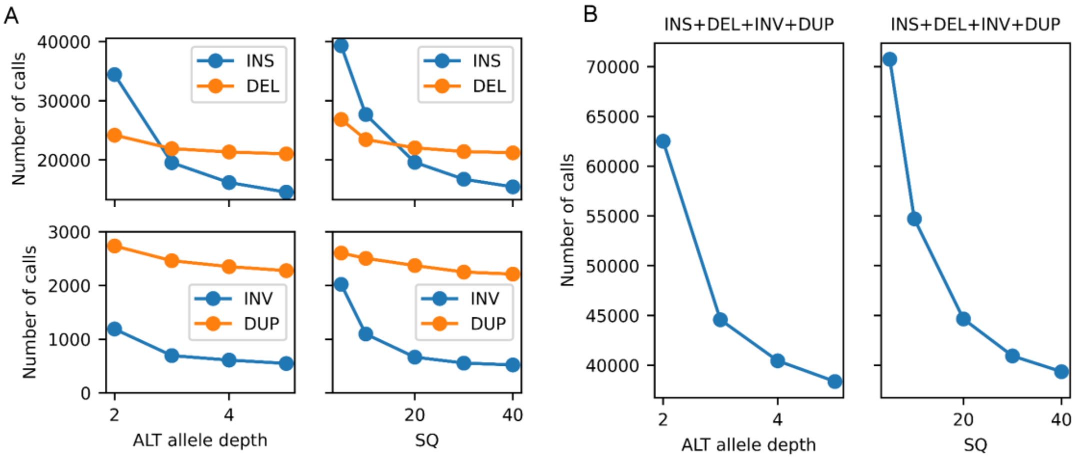

Figure S16. Filtering non-TR SVs with allele depth (AD) and sample quality (SQ). Related to STAR methods.
(A) Number of calls stratified by SV type. **(B)** Total number of SVs.

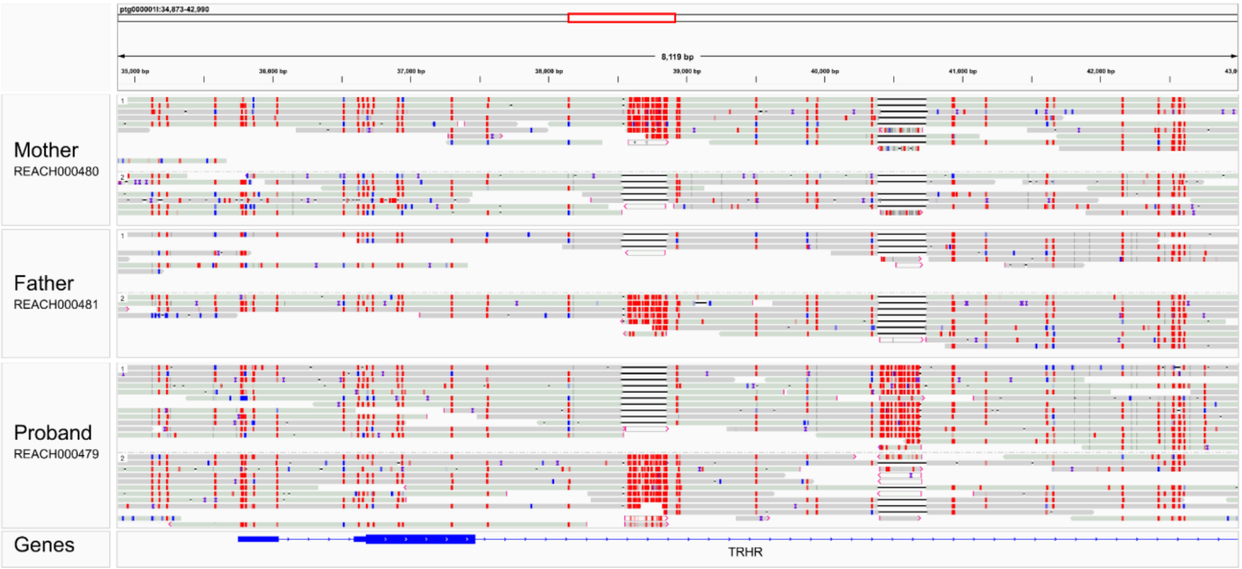

Figure S17. Methylation signature of a common *Alu* insertion and a private *de novo Alu* insertion in the *TRHR* intron. Related to STAR methods.
Long reads from a trio are mapped to an assembled contig containing *TRHR* (86kb). The assembled contig is constructed based on the maternal haplotype of the proband (REACH000479). The *de novo* Alu sequence from the paternal haplotype of the same subject is merged to this contig to include both Alu sequences in the contig. H1 in the mother, H2 in the father and H2 in the proband have the left *Alu*, a common variant in the population, while the *de novo* Alu on the right is present only in the proband. The methylation likelihood of the CpG sites are colored with red indicating high methylation likelihood. As expected, the *Alus* contain CpG sites mostly methylated.

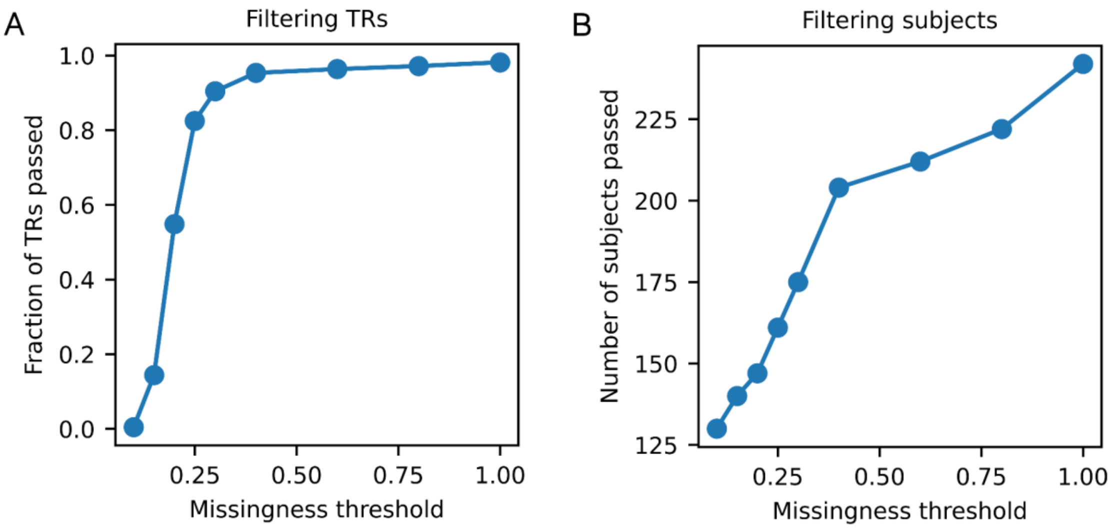

Figure S18. Filtering TR regions and subjects as a function of missingness. Related to STAR methods.
(A) Fraction of TR regions passed as a function of missingness threshold. Missingness for a TR region is defined as the fraction of subjects missing genotypes for the TR region. **(B)** Number of subjects passed as a function of missingness threshold. Missingness for a subject is defined as the fraction of TRs not genotyped for the subject.

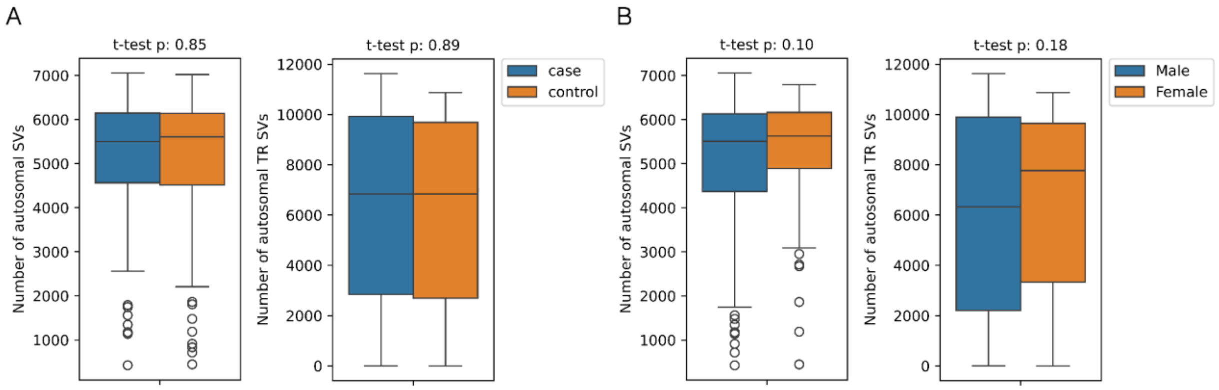

Figure S19. Number of autosomal SVs and TR-SVs stratified by case status and sex. Related to STAR methods.
The number of autosomal SVs and TRs is not significantly different when stratified by case status and sex of the individuals in the cohort.

[S1] Akbari, V., Dada, S., Shen, Y., Dixon, K., Hejla, D., Galbraith, A., Choufani, S., Weksberg, R., Boerkoel, C.F., Stewart, L., et al. (2024). Long-read sequencing for detection and subtyping of Prader-Willi and Angelman syndromes. J. Med. Genet. *62*, 32–36.

[S2] Jima, D.D., Skaar, D.A., Planchart, A., Motsinger-Reif, A., Cevik, S.E., Park, S.S., Cowley, M., Wright, F., House, J., Liu, A., et al. (2022). Genomic map of candidate human imprint control regions: the imprintome. Epigenetics *17*, 1920–1943.
